# Reflecting on what is “skill” in human motor skill learning

**DOI:** 10.3389/fnhum.2023.1117889

**Published:** 2023-07-06

**Authors:** Goldy Yadav, Julie Duque

**Affiliations:** Cognition and Actions Lab, Institute of Neuroscience, Université Catholique de Louvain, Brussels, Belgium

**Keywords:** motor skills, learning, motor control, skill behavior, movements, motor errors

## Abstract

Humans have an exceptional ability to execute a variety of skilled movements. Researchers have been long interested in understanding behavioral and neurophysiological basis of human motor skill learning for advancing both fundamental neuroscientific knowledge and clinical outcomes. However, despite decades of work in this field there is a lack of consensus about what is meant by “skill” in skill learning. With an advent of various task paradigms testing human motor behavior and increasing heterogeneity in motor learning assessments methods, it is very crucial to identify key features of skill in order to avoid any ambiguity that may result in misinterpretation or over-generalization of findings, which could have serious implications for replication and translational research. In this review, we attempt to highlight the features of skill following a historical approach, considering the seminal work that led to the first definitions of skill and including some contemporary concepts emerging from human motor learning research. Overall, based on this literature, we emphasize that skill has some fundamental characteristics, such as- (i) optimal movement selection and execution, (ii) improved movement speed and accuracy, and (iii) reduced movement variability and error. These features of skill can emerge as a consequence of extensive practice/training/learning, thus resulting in an improved performance state beyond baseline levels. Finally we provide some examples of model tasks that can appropriately capture these features of skill, and conclude that any neuroscientific endeavor aimed at understanding the essence of skill in human motor skill learning should focus on these aspects.

## 1. Introduction

Human beings can learn to perform a variety of skilled movements ranging from dancing, painting, playing musical instruments, riding a bicycle, driving a car, playing a wide range of sports and so on. This ability to perform such complex movements is undoubtedly one of the characteristic functions of the human brain which enables us to interact with our environment. Even simpler actions such as reaching for and grasping a cup of tea, holding a pen for writing and tying one’s shoelaces are complex problems for the nervous system, but are performed in a rather seamless manner by us on a day-to-day basis. Motor skill learning is a key ability that enables us to acquire and store such actions and distinguishes humans from other species. As a society we value this ability which enables us to make a wide variety of movements, and are awed by the fine skills of musicians, dancers and sports players. Scientists have long been fascinated by this capacity of humans to learn complex skilled movements and have tried to probe its underlying neurophysiological basis to advance fundamental neuroscientific understanding as well as for translational goals.

Literature available on skill learning can sometime confuse readers and/or young researchers getting into this field of study. As a master’s student doing her thesis on skill learning and retention in 2014, the now co-author of this paper (Goldy Yadav) often came across research papers loosely using the term “skill” for tasks assessing motor performance under controlled conditions (such as motor learning under externally induced perturbations). Despite several decades of work, we still lack a clear understanding of what we mean by skill in human motor learning research field. Challenges in formulating a formal definition of skill has been felt by many, as our views are fragmented and lack consensus (nicely elucidated more recently by Christensen, 2019). This can have serious implication for result interpretations and replication of findings in this area of research. Moreover, this can be very misleading not only for fundamental motor skill learning research, but also motor rehabilitation programs which aim to translate lab-based research findings for patient benefits. A much-needed consensus on definitions and measurement standards of task paradigms is critical for reducing the time lag in translation of scientific discoveries for clinical practices (Shumway-Cook and Woollacott, 2007; Morris et al., 2011).

This review paper is therefore an attempt to obtain clarity on what is meant by “skill” by highlighting key concepts that came out of some seminal work and how those ideas can be used as a strong framework for current and future skill learning research. We aim to shed light on how our understanding of skill in the context of human motor learning has evolved with time. First, we lay out what is broadly meant by motor learning and highlight early theories of motor learning that shaped our understanding of skills. Next, we delve deeper into a specific form of motor learning that is of interest to us, i.e., motor skill learning and attempt to understand the specific features of skill based on prevailing research in the field. Finally, we conclude by emphasizing the key features of skill that should be incorporated in motor skill learning research work to better assess, measure and understand the underlying nature of human skill behavior. We also provide some examples of existing model tasks that very well incorporate these features.

## 2. What is motor learning?

Motor learning can be broadly defined as practice induced changes in motor performance. The early era of motor learning research involved studying motor behavior using a “task oriented” approach and the term “skill” was often synonymous with perceptual-motor performance. At that time there was little emphasis on variables or factors that underlie motor learning at an individual level. Such work explored global features of motor learning in humans (Hull, 1943), and often involved gross measurements and scoring of movement parameters such as movement time. Over time, researchers started shifting from these global measures to local measures in order to better understand the nuances of human motor behavior. Some of these assessments involved studying the amount of time taken to process visual information before initiating movements, the role of attention in motor performance and how error detection occurs during the movement (Schmidt, 1975). As a result, a number of theories were proposed to explain human motor learning. For instance, Keele (1968) proposed that “motor programs” are required for making precise and accurate bodily movements. These motor programs were thought of as a sequence of “stored commands” in the brain which enables us to make a set of movements by seamlessly incorporating the external feedback. Later Adams (1971) argued that motor programs are abstract memory forms that are prepared before movement initiation and contain information about the pattern of muscle contraction and relaxation for a given movement type. In other words, the human brain acquires and stores a set of stored muscle commands that are ready to use at any given time for making a movement.

Around the same time period, another popular theory about motor learning was proposed by Schmidt (1975)- this theory was an attempt to explain why we need not have distinct individual motor commands for each movement that we make. According to this theory, there are clustered and generalized motor programs called “schemas,” which can be fine-tuned to make specific discrete sets of movements. In fact, long ago Bartlett (1932) had suggested that such schemas are critical given the limited storage capacity of the human brain, and possibly underlie our astonishing ability to quickly learn novel sets of movements. Interestingly, the idea of schema formation underlying motor learning is evident in some recent work as well (Newell, 2003; Sherwood and Lee, 2003; Shea and Wulf, 2005; van Vugt et al., 2014; Willey and Liu, 2018). This notion has also been used to explain motor skill behavior. For instance, van Vugt et al. (2014) studied expert pianists and found that highly skilled complex actions emerge from a combination of simpler movements known as motor primitives (equivalent of motor schema). The authors argue that our nervous system efficiently uses these motor primitives to produce a wide repertoire of complex movements that can be enhanced through skill learning. We believe these concepts of skill can also have implications for studying and understanding expert motor skill behavior which typically operates on a different timescale than lab-based experimental setup requiring hundreds of hours of training, but may share underlying common features (for interesting work on elite performers see Wulf et al., 2002; Beilock and Carr, 2004; Wulf and Su, 2007; Abdollahipour et al., 2015; Singh and Wulf, 2022).

According to the schema model of motor learning (Schmidt, 1975), the nervous system requires four parameters for a goal-directed movement–(i) initial task conditions, (ii) response specifications of the movement to be made, (iii) sensory consequences of the executed movement, and (iv) outcome of the executed movement. Eventually after producing a set of movements, the nervous system extracts abstract information about the relationship among these four parameters. This abstract relationship is stored as schema to enable us to perform these movements later when required. Once a schema has developed, only two inputs/specifications are needed to this schema in order to execute a movement- (i) initial task conditions and, (ii) the desired outcome of the movement. Schmidt (1975) argued that since these inputs to the schema are never exactly the same, every movement that we execute is almost always novel, although appearing similar from a broader level. This view finds support from Bartlett (1932) who said, “we do not execute the movement exactly as we have made it before.” This may explain the inherent variability underlying complex movement execution (more details on variability in Section “3.3. Skill as performance optimization”). Basically, a motor response is nearly never repeated given the number of possibilities that may arise from a given schema. In this process of motor execution, sensory outcomes of the movement are anticipated using the schema and each of the resultant sensory outcomes are compared with respective incoming sensory information (interoceptive and proprioceptive feedback) during or after the movement. In case of a mismatch between the anticipated and actual sensory information, an error value is assigned which is later used to update the schema. The primary goal of the nervous system, therefore, during motor learning is to reduce such errors.

The errors resulting due to the mismatch between anticipated and actual sensory consequences can also serve as a substitute for knowledge of results (KR) which can be used to update the schema (Salmoni et al., 1984; Winstein, 1991; Guadagnoli and Kohl, 2001). KR is one of the key ways to improve motor performance and enhance learning. During a motor learning task, KR is mostly presented in three ways so as to facilitate corrective responses (Adams, 1987)–(i) by directly presenting the pattern of the motor response to the participant for which error must be inferred, (ii) feedback in the form of the actual motor response that the participant made along with the ideal expected motor response, where the difference between the two is the error which can be easily inferred by the participant; and (iii) partial error information for the motor response made by the participant. In addition to providing KR to improve motor performance, learning by observation is another effective way. Newell (1985) proposed that learning through observations may help in easy identification of errors, and hence enable efficient motor responses. Motor learning by observation has been mainly shown to enhance performance in a number of studies (Shea et al., 2000; Stefan et al., 2005; Wulf et al., 2005; Celnik et al., 2006; Granados and Wulf, 2007; Nishizawa and Kimura, 2017; Kawasaki et al., 2018; Jayasinghe, 2019), with some distinctive effects reported for observation and motor task type (please see Sorgente et al., 2022 for details). KR and observation, therefore, may play a critical role (as external factors/interventions) during skill acquisition to optimize skill performance (more details in section “3.3. Skill as performance optimization”).

**FIGURE 1:**
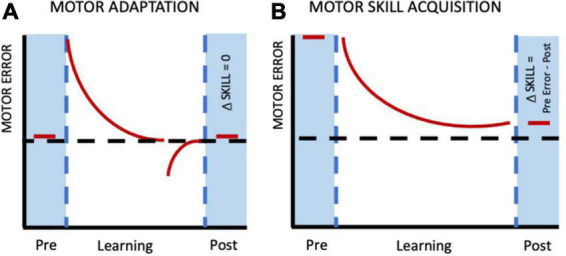
Difference between motor adaptation and motor skill learning. **(A)** In motor adaptation pre-perturbed behavior is re-established and therefore, no net change in the motor performance per se as compared to the baseline/starting level. **(B)** During skill acquisition, there are performance improvements (requiring longer time period) beyond baseline levels leading to a net change in motor behavior (pre-vs. post-learning). Adapted from Chapter 8, Sternad et al. (2014).

The points described above highlight the broader aspects of human motor learning. Now let’s focus more specifically on how motor learning is studied in the laboratory. In this context it is largely investigated using two classic paradigms- ***motor skill learning*** and ***motor adaptation***. Our understanding of each of these two paradigms has evolved with time, enabling us to better understand the nuances of human motor behavior. Motor skill learning is characterized by performance changes beyond baseline/starting levels in the absence of any external perturbation (more details in the next section). Such changes may be characterized, for instance, by increased spatial and temporal accuracy of movements over the practice session (Adams, 1987; Reis et al., 2009; van der Steen et al., 2014; Yadav and Mutha, 2020). Features of skill learning include, but are not limited to- reduction in trial-to-trial variability, changes in speed-accuracy relationship or other performance limiting variables, offline gains, acquisition of new control policies and exploration (Dayan and Cohen, 2011; Shmuelof et al., 2012; Telgen et al., 2014; Sternad, 2018; Vassiliadis et al., 2021; Du et al., 2022). In contrast, motor adaptation involves modification of motor output to account for the effects of externally induced perturbations and restoration of performance to pre-perturbation levels (Krakauer and Mazzoni, 2011; Morehead et al., 2017; Kumar et al., 2020). Figure 1 (Adapted from Chapter 8, Sternad et al., 2014) shows clear distinction between motor adaptation and motor skill learning. As evident from the figure, the goal during motor adaptation is to return to baseline performance (low motor error) by reducing the errors arising from external perturbations. On the other hand, skill learning involves gradual reduction of errors that are high in the beginning of the session because of the novel nature of the movements and the need to acquire a new control policy. Skill acquisition involves de novo learning in contrast to adaptation (Sternad, 2018; Krakauer et al., 2019).

In addition to these behavioral differences, adaptation and skill learning are also thought to be dependent on distinct neural systems. For instance, motor skill learning appears to be primarily mediated via primary motor cortex and basal ganglia circuits (Floyer-Lea and Matthews, 2005; Halsband and Lange, 2006; Doyon et al., 2009; Dayan and Cohen, 2011; Cantarero et al., 2013; Spampinato and Celnik, 2018) while adaptation seems to requires an intact cerebellum and posterior parietal cortex (Tseng et al., 2007; Mutha et al., 2011a,b; Morehead et al., 2017; Kumar et al., 2020). It is therefore critical to keep these differences between motor skill learning and adaptation in mind while formulating research questions on the nature of human motor behavior to avoid any ambiguity and over-generalization across paradigms (Ranganathan et al., 2021). Further, understanding these distinctions also help exercise caution during data interpretation.

In this paper, we focus on motor skill learning to unravel the characteristic features of skill. With this goal in mind, we review the prevailing ideas of what is meant by “skill” in this field of study. We are specifically interested in skill learning because it holds tremendous implications for motor rehabilitation- complex motor skill behavior in humans and its rehabilitation following neurological damage are critically dependent on the ability to learn and regain lost motor skills. We therefore believe that it is very crucial to have some consensus and clarity on what we mean by “skill.” Adopting clear working definition(s) of motor skills is important when our collective goal is to better understand human skill behavior.

## 3. What are motor skills?

The ability to execute and learn a variety of skilled movements is an astonishing human feat. Skill emerges as a consequence of learning/training/extensive practice. Azim (2014) said- “Few of our limb movements will ever gain immortality like Willie Mays, but what we accomplish every day is remarkable.” Study of human motor skills is of tremendous neuroscientific interest, and several attempts have been made to define motor skills over these years. Pear (1927) loosely defined skill as well-adjusted and integrated motor performance, which is dependent on learning and optimal motor output, and therefore distinct from a mere capacity to perform a given motor task. Later in 1952, Guthrie stated that motor skill is an ability to perform with high certainty that require minimum energy and time. Willingham (1998) proposed a neurophysiological theory of motor skill learning in which he describes that skill learning emerges out of motor control processes. While motor control involves planning and execution of movements (Hommel, 2009; Krakauer et al., 2019; Merel et al., 2019), motor skill learning, in addition to involving movement selection and execution, is the process through which movement quality is improved with practice (Adams, 1987; Willingham, 1998; Dayan and Cohen, 2011; Chen et al., 2018; Sternad, 2018).

### 3.1. Control based learning theory of motor skills

According to Willingham (1998), there are three motor control processes underlying motor skills- (i) perceptual-motor integration which involves selection of spatial targets for movement, (ii) processing target features, and (iii) transforming these target features into desired muscle/motor commands (dynamic processing) to execute the skilled movement. These control processes are fine-tuned depending on the task specificity and requirements and these operate in an unconscious manner to improve motor performance. In addition, Willingham also added a fourth component (iv) which involves using strategies (operating in “conscious” manner) to further enhance performance outcomes on a motor task, an idea that is now being supported by several authors (Wulf et al., 2001, 2010; Fridland, 2014; Christensen, 2019; Yadav and Mutha, 2020).

Willingham (1998) attempted to present a comprehensive framework of motor skill learning by including all these processes in the form of COBALT, i.e., Control Based Learning Theory. COBALT comprises of three key principles. First principle is “neural separability principle” according to which each component of motor skill processing is mediated by distinct neural areas. For instance, strategic-conscious processes are mediated by Dorsolateral Prefrontal Cortex (DLPFC), perceptual-motor integration is mediated by posterior parietal and pre-motor cortical regions, target processing and planning by supplementary motor area and basal ganglia, and dynamic processing by spinal cord (spinal interneurons). Several studies support such a view of an engagement of multiple brain areas in mediating human motor skill behavior (Poldrack et al., 1998, 2005; Van Horn et al., 1998; Poldrack and Gabrieli, 2001; Penhune and Doyon, 2002; Reis et al., 2009; Dayan and Cohen, 2011). The second principle of COBALT framework is “disparate representation principle.” According to this, each of the four motor control processes (mentioned in the previous paragraph) utilize distinct forms of representations. Willingham (1998) proposed that strategic-conscious processes are represented in allocentric (extrinsic world or object-centered) space. The other three, i.e., perceptual-motor integration, target processing and dynamic processing rely on egocentric (intrinsic body-centered) space. This notion again finds support in numerous studies of motor learning (Hikosaka et al., 1999; Criscimagna-Hemminger et al., 2003; Lange et al., 2004). It is also believed that these two forms of representations, viz., object-centered and body-centered, have important implications for motor skill learning- the former mediates more effector-independent representations whereas the latter mediates effector-dependent representations (Colby and Goldberg, 1999; Hikosaka et al., 1999; Krakauer et al., 1999; Lange et al., 2004; Boutin et al., 2012). In other words, skill can have both effector-dependent and effector-independent components. Finally, the third principle of COBALT is “dual mode principle,” which proposes two modes for operation for these four motor control processes- conscious and unconscious modes. The strategic and target processing are mediated by the conscious mode whereas the other two factors, namely perceptual-motor integration and dynamic processing are mediated by the unconscious mode.

Willingham (1998) COBALT theory is, therefore, an all-encompassing attempt to explain skilled motor behavior through motor control processes. However, this theory is mostly centered around target information, which is mostly about spatial accuracy, and therefore lacks any account of temporal aspects of skill, which we now know as a key component of motor skill experiments in the lab (Kantak et al., 2010; Shmuelof et al., 2012; Yadav and Mutha, 2016, 2020; Vassiliadis et al., 2021, 2022). Including temporal accuracy as one of the key components of motor skill (in addition to spatial accuracy as already elaborated by Willingham, 1998) is needed while investigating and assessing human skill performance. Such components (for instance, spatial accuracy and temporal accuracy) will serve to optimize skill behavior (by inducing task constrains) and will help us better understand what constitutes a highly skilled performance.

### 3.2. Skill as a reflection of a change in speed-accuracy relationships

Task success on a novel motor skill is highly constrained by difficulty levels which can arise from trying to optimize various task parameters during initial stages of learning. Hence, the main goal in performing and learning a skilled movement involves an ability to make fast and accurate movements simultaneously. This requires overcoming the trade-off between speed and accuracy which are inherently constraint inducing factors in a motor skill task (Reis et al., 2009; Shmuelof et al., 2012; Yadav and Mutha, 2016, 2020; Chen et al., 2018; Vassiliadis et al., 2021, 2022). Speed-accuracy relationship has thus emerged as a key feature of skill learning in humans.

Changes in speed-accuracy relationship is, therefore, a global feature of motor skill learning mediated by kinematic level changes such as reduced variability, increased movement smoothness and speed that overall results in performance improvements and task success (Shmuelof et al., 2012; Telgen et al., 2014). Even though there is no formal definition of skill learning, across all major research studies it is mainly characterized by improved movement quality, reduction in trial-to-trial variability, increased movement smoothness, changes in speed-accuracy relationships (Shmuelof et al., 2012; Telgen et al., 2014), offline gains/improvements (Walker et al., 2002; Dayan and Cohen, 2011) and acquisition of new control policies or strategies (Telgen et al., 2014; Yadav and Mutha, 2020) to improve task success (Shmuelof et al., 2012). Out of all these, change in speed-accuracy relationship is probably a global process that underlies motor skill learning in humans because a highly accurate but slow movement or a fast but less accurate movement will not yield a skilled movement that we observe in humans (dance, sports, playing musical instrument and so on). This is in line with Guthrie’s idea (Guthrie, 1952) we stated earlier relating skill to time and energy efficient performance. Skill behavior, we believe, is therefore a result of performance optimization involving overcoming performance limiting variables such as speed, accuracy and motor variability (noise).

### 3.3. Skill as performance optimization

During acquisition of novel motor skills, one of the main goals is to reduce the movement error and the variability in the error (Deutsch and Newell, 2004; Müller and Sternad, 2004; Shmuelof et al., 2012; Telgen et al., 2014); while also increasing movement speed, accuracy and efficiency (Willingham, 1998; Schmidt and Lee, 2005). Sternad (2018) argues that although mastering a new skill involves diminishing variability or noise in motor performance, variability plays an important role in exploring successful action(s) in the initial stages of learning a novel skill task. Given the redundancy at the level of movement outcome, variability in that context may help a learner to find stable solutions that reduce performance errors. For instance, Pekny et al. (2015) showed in their study that healthy individuals increase trial-to-trial variability to make corrections while making fast reaching movements in the absence of any reward. It is important to note that such movement variability may also exist at the level of movement execution without covertly affecting movement outcome (Todorov and Jordan, 2002; Müller and Sternad, 2009). Thus, high movement variability in the early stages of learning can be beneficial for skill acquisition (exploration of task space for finding optimal movement), while the goal of the nervous system may still be to actively minimize it as learning progresses (Fitts, 1954; Telgen et al., 2014).

Another challenge during skill acquisition is that there is no predetermined performance limit to aim for and, therefore the ideal performance cannot be planned or predicted in advance, yet the features of the acquired skill are somewhat constrained by the external environment and in the context of the lab setups, by the experimental designs and data collection/analysis procedures. In other words, even if constrained by the environment, each individual has to “figure out” the ideal skill performance during the course of practice/training. Moreover, there is a redundancy at the level of motor execution which adds additional burden of choosing the optimal movement from a wide repertoire of feasible movements. This ability to choose the most optimal movement from a vast set of movements forms an integral part of skilled motor behavior. Thorndike and and Columbia University, Institute of Educational Research, Division of Psychology (1932) proposed that the goal for an individual is to choose the action that gives maximum reward over the ones that result in errors. This requires exploring the optimal solution during learning probably through trial-and-error, and for that extensive practice is very beneficial. Müller and Sternad (2004) used a virtual skittles task to study skill acquisition and demonstrated that individuals are able to learn the optimal solution that maximizes task accuracy after a substantial amount of practice. Similarly, Shmuelof et al. (2012) studied skill acquisition on a motor task that involved wrist flexion-extension and pronation-supination in an arc channel, and examined changes in speed-accuracy tradeoff with practice and the underlying kinematic improvements in performance. After 5 days of training the authors noted that participants demonstrated large reduction in movement variability, increase in movement smoothness and a shift in speed-accuracy tradeoff. This ability to improve performance by changing speed-accuracy tradeoff, which was later termed “motor acuity” by Shmuelof et al. (2012, 2014), is now considered a key feature of motor skill behavior (Krakauer et al., 2019; Du et al., 2022). To sum up, skill is a state of optimized performance that rely on extensive practice/training/learning to be able to select and execute a movement with high speed and accuracy thus resulting in low errors and variability on a given motor task.

## 4. Conclusion

**FIGURE 2:**
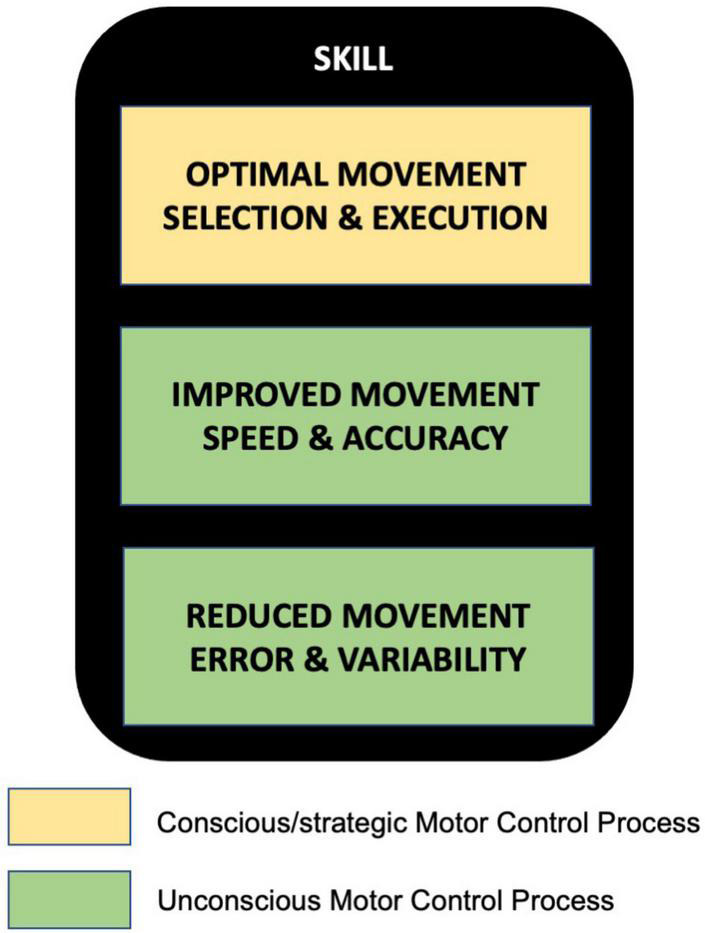
Key features of skill in human motor skill learning-(i) Optimal movement selection and execution, (ii) Improved movement speed and accuracy, and (iii) Reduced movement error and variability. The first feature may be mediated by conscious/strategic motor control processes, whereas the other two by unconscious motor control processes.

A key form of human motor behavior is skill learning, which is distinct from another popular task paradigm known as motor adaptation. Characterizing key features of motor skills, we believe, is the first critical step toward understanding how humans acquire this quintessential ability through training. Based on prior work by other researchers outlined in this paper, it appears that motor skill can be broadly characterized by three key features (Figure 2)- (i) optimal movement selection and execution, (ii) improved movement speed and accuracy, and (iii) reduced movement variability and error, as a consequence of extensive practice. Here, the first feature may rely on external cues such as target information and thus primarily involve strategic/conscious motor control processes in the early stages of skill acquisition. The latter two, on the other hand, may mainly be driven by unconscious processes that help overcome task constraints resulting in a performance state beyond baseline level. A culmination of these aspects, we believe, finally results in a seamless automatic skill behavior that we observe in humans, which may further evolve as expert-level performances (with extensive practice/training) in the form of dancing, painting, playing musical instruments, sports, and so on.

The three key features mentioned above, therefore, should be included in any task designed to capture and study motor skills. For example, lab based experimental procedures of skill learning (wherein extensive practice spanning months and/or years is not possible) can exclusively assess individuals on motor tasks with a given speed-accuracy condition that requires them to acquire optimal movement (from a repertoire of possible movements) leading to low error/variability. Skill tasks that very well capture these aspects are, for instance, those that involve tracing movements within a given speed-accuracy criterion and pinch-based movements requiring optimal force production to make an accurate goal-directed movement. In addition to these, even point-to-point reaching movements that involve moving the effector end-points as well as the joints (elbow and shoulder for instance) in a manner that reduces movement-related errors toward a target (in the absence of any external perturbation) could be a simple skill task to use in the lab (especially for patient populations with motor disability). Such task setups and assessments can capture the most fundamental aspect of human motor skill behavior which involves selection and execution of movements that optimize time and energy (internally) while yielding successful purposeful outcomes (externally). Hence, systematic use of such tasks would ensure homogeneity at the methodological level, help build more comprehensive theoretical framework and speed up cumulative progress of human motor skill research. Further, the resultant clarity would help curb replication crisis in human motor behavior research and benefit translation of fundamental knowledge to clinical settings- both relying primarily on agreed upon measures and definitions for scientific knowledge enhancement and knowledge transfer.

## Author contributions

Both authors listed have made a substantial, direct, and intellectual contribution to the work, and approved it for publication.

## References

[B1] Abdollahipour R. Wulf G. Psotta R. Palomo Nieto M. (2015). Performance of gymnastics skill benefits from an external focus of attention. *J. Sports Sci.* 33 1807–1813. 25774536 10.1080/02640414.2015.1012102

[B2] Adams J. A. (1971). A closed-loop theory of motor learning. *J. Motor Behav.* 3 111–150. 10.1080/00222895.1971.10734898 15155169

[B3] Adams J. A. (1987). Historical review and appraisal of research on the learning, retention, and transfer of human motor skills. *Psychol. Bull.* 101 41–74.

[B4] Azim E. (2014). Shortcuts and checkpoints on the road to skilled movement. *Science* 346, 554–555. 25359954 10.1126/science.1260778

[B5] Bartlett F. C. (1932). *Remembering: An experimental and social study.* Cambridge, MA: Cambridge University.

[B6] Beilock S. L. Carr T. H. (2004). “From novice to expert performance: Memory, attention and the control of complex sensori-motor skills,” in *Skill acquisition in sport*, ed. Shields T. (Oxfordshire, UK: Routledge), 333–351.

[B7] Boutin A. Badets A. Salesse R. N. Fries U. Panzer S. Blandin Y. (2012). Practice makes transfer of motor skills imperfect. *Psychol. Res.* 76 611–625. 10.1007/s00426-011-0355-2 21671102

[B8] Cantarero G. Lloyd A. Celnik P. (2013). Reversal of long-term potentiation-like plasticity processes after motor learning disrupts skill retention. *J. Neurosci.* 33 12862–12869. 10.1523/JNEUROSCI.1399-13.2013 23904621 PMC3728692

[B9] Celnik P. Stefan K. Hummel F. Duque J. Classen J. Cohen L. G. (2006). Encoding a motor memory in the older adult by action observation. *Neuroimage* 29 677–684. 16125417 10.1016/j.neuroimage.2005.07.039

[B10] Chen X. Holland P. Galea J. M. (2018). The effects of reward and punishment on motor skill learning. *Curr. Opin. Behav. Sci.* 20 83–88.

[B11] Christensen W. (2019). Skilled action. *Philos. Compass* 14:e12631.

[B12] Colby C. L. Goldberg M. E. (1999). Space and attention in parietal cortex. *Annu. Rev. Neurosci.* 22 319–349. 10202542 10.1146/annurev.neuro.22.1.319

[B13] Criscimagna-Hemminger S. E. Donchin O. Gazzaniga M. S. Shadmehr R. (2003). Learned dynamics of reaching movements generalize from dominant to nondominant arm. *J. Neurophysiol.* 89 168–176. 12522169 10.1152/jn.00622.2002

[B14] Dayan E. Cohen L. G. (2011). Neuroplasticity subserving motor skill learning. *Neuron* 72 443–454. 22078504 10.1016/j.neuron.2011.10.008 PMC3217208

[B15] Deutsch K. M. Newell K. M. (2004). Changes in the structure of children’s isometric force variability with practice. *J. Exp. Child Psychol.* 88 319–333. 15265679 10.1016/j.jecp.2004.04.003

[B16] Doyon J. Bellec P. Amsel R. Penhune V. Monchi O. Carrier J. (2009). Contributions of the basal ganglia and functionally related brain structures to motor learning. *Behav. Brain Res.* 199 61–75. 19061920 10.1016/j.bbr.2008.11.012

[B17] Du Y. Krakauer J. W. Haith A. M. (2022). The relationship between habits and motor skills in humans. *Trends Cogn. Sci.* 26 371–387. 35307293 10.1016/j.tics.2022.02.002

[B18] Fitts P. M. (1954). The information capacity of the human motor system in controlling the amplitude of movement. *J. Exp. Psychol.* 47:381. 13174710

[B19] Floyer-Lea A. Matthews P. M. (2005). Distinguishable brain activation networks for short- and long-term motor skill learning. *J. Neurophysiol.* 94 512–518. 10.1152/jn.00717.2004 15716371

[B20] Fridland E. (2014). They’ve lost control: Reflections on skill. *Synthese* 191 2729–2750.

[B21] Granados C. Wulf G. (2007). Enhancing motor learning through dyad practice: Contributions of observation and dialogue. *Res. Q. Exerc. Sport* 78 197–203. 10.1080/02701367.2007.10599417 17679493

[B22] Guadagnoli M. A. Kohl R. M. (2001). Knowledge of results for motor learning: Relationship between error estimation and knowledge of results frequency. *J. Motor Behav.* 33 217–224. 10.1080/00222890109603152 11404216

[B23] Guthrie E. R. (1952). *The psychology of learning, Rev.* New York, NY: Harper.

[B24] Halsband U. Lange R. K. (2006). Motor learning in man: A review of functional and clinical studies. *J. Physiol. Paris* 99 414–424. 10.1016/j.jphysparis.2006.03.007 16730432

[B25] Hikosaka O. Nakahara H. Rand M. K. Sakai K. Lu X. Nakamura K. (1999). Parallel neural networks for learning sequential procedures. *Trends Neurosci.* 22 464–471. 10481194 10.1016/s0166-2236(99)01439-3

[B26] Hommel B. (2009). Action control according to TEC (theory of event coding). *Psychol. Res. PRPF* 73 512–526. 10.1007/s00426-009-0234-2 PMC2694931 19337749

[B27] Hull C. L. (1943). *Principles of behavior (Vol. 422).* New York, NY: Appleton-century-crofts.

[B28] Jayasinghe S. A. L. (2019). The role of sensory stimulation on motor learning via action observation: A mini review. *J. Neurophysiol.* 121 729–731. 10.1152/jn.00747.2018 30517045

[B29] Kantak S. S. Sullivan K. J. Fisher B. E. Knowlton B. J. Winstein C. J. (2010). Neural substrates of motor memory consolidation depend on practice structure. *Nat. Neurosci.* 13 923–925. 10.1038/nn.2596 20622872

[B30] Kawasaki T. Tozawa R. Aramaki H. (2018). Effectiveness of using an unskilled model in action observation combined with motor imagery training for early motor learning in elderly people: A preliminary study. *Somatosens. Motor Res.* 35 204–211. 10.1080/08990220.2018.1527760 30592442

[B31] Keele S. W. (1968). Movement control in skilled motor performance. *Psychol. Bull.* 70:387.

[B32] Krakauer J. W. Mazzoni P. (2011). Human sensorimotor learning: Adaptation, skill, and beyond. *Curr. Opin. Neurobiol.* 21 636–644. 21764294 10.1016/j.conb.2011.06.012

[B33] Krakauer J. W. Ghilardi M. F. Ghez C. (1999). Independent learning of internal models for kinematic and dynamic control of reaching. *Nat. Neurosci.* 2 1026–1031. 10526344 10.1038/14826

[B34] Krakauer J. W. Hadjiosif A. M. Xu J. Wong A. L. Haith A. M. (2019). Motor learning. *Compr. Physiol.* 9 613–663. 30873583 10.1002/cphy.c170043

[B35] Kumar A. Panthi G. Divakar R. Mutha P. K. (2020). Mechanistic determinants of effector-independent motor memory encoding. *Proc. Natl. Acad. Sci. U. S. A.* 117 17338–17347. 10.1073/pnas.2001179117 32647057 PMC7382293

[B36] Lange R. K. Godde B. Braun C. (2004). EEG correlates of coordinate processing during intermanual transfer. *Exp. Brain Res.* 159 161–171. 15340766 10.1007/s00221-004-1942-x

[B37] Merel J. Botvinick M. Wayne G. (2019). Hierarchical motor control in mammals and machines. *Nat. Commun.* 10:5489. 10.1038/s41467-019-13239-6 PMC6889345 31792198

[B38] Morehead J. R. Taylor J. A. Parvin D. E. Ivry R. B. (2017). Characteristics of implicit sensorimotor adaptation revealed by task-irrelevant clamped feedback. *J. Cogn. Neurosci.* 29 1061–1074. 10.1162/jocn_a_01108 28195523 PMC5505262

[B39] Morris Z. S. Wooding S. Grant J. (2011). The answer is 17 years, what is the question: Understanding time lags in translational research. *J. R. Soc. Med.* 104 510–520. 10.1258/jrsm.2011.110180 22179294 PMC3241518

[B40] Müller H. Sternad D. (2004). Decomposition of variability in the execution of goal-oriented tasks: Three components of skill improvement. *J. Exp. Psychol.* 30:212. 10.1037/0096-1523.30.1.212 14769078

[B41] Müller H. Sternad D. (2009). “Motor learning: Changes in the structure of variability in a redundant task,” in *Progress in motor control: A multidisciplinary perspective*, ed. Sternad D. (Berlin: Springer), 439–456. 10.1007/978-0-387-77064-2_23 PMC3776417 19227514

[B42] Mutha P. K. Sainburg R. L. Haaland K. Y. (2011a). Critical neural substrates for correcting unexpected trajectory errors and learning from them. *Brain* 134 3647–3661. 10.1093/brain/awr275 22075071 PMC3235559

[B43] Mutha P. K. Sainburg R. L. Haaland K. Y. (2011b). Left parietal regions are critical for adaptive visuomotor control. *J. Neurosci.* 31 6972–6981. 21562259 10.1523/JNEUROSCI.6432-10.2011 PMC3107546

[B44] Newell K. M. (1985). Coordination, control and skill. *Adv. Psychol.* 27 295–317.

[B45] Newell K. M. (2003). Schema theory (1975): Retrospectives and prospectives. *Res. Q. Exerc. Sport* 74 383–388. 10.1080/02701367.2003.10609108 14768839

[B46] Nishizawa H. Kimura T. (2017). Enhancement of motor skill learning by a combination of ideal model-observation and self-observation. *J. Phys. Ther. Sci.* 29 1555–1560. 10.1589/jpts.29.1555 28931987 PMC5599820

[B47] Pear T. H. (1927). Skill. *J. Pers. Res*. 5, 478–489.

[B48] Pekny S. E. Izawa J. Shadmehr R. (2015). Reward-dependent modulation of movement variability. *J. Neurosci.* 35 4015–4024. 25740529 10.1523/JNEUROSCI.3244-14.2015 PMC4348194

[B49] Penhune V. B. Doyon J. (2002). Dynamic cortical and subcortical networks in learning and delayed recall of timed motor sequences. *J. Neurosci.* 22 1397–1406. 10.1523/JNEUROSCI.22-04-01397.2002 11850466 PMC6757579

[B50] Poldrack R. A. Gabrieli J. D. (2001). Characterizing the neural mechanisms of skill learning and repetition priming: Evidence from mirror reading. *Brain* 124 67–82. 10.1093/brain/124.1.67 11133788

[B51] Poldrack R. A. Desmond J. E. Glover G. H. Gabrieli J. D. (1998). The neural basis of visual skill learning: An fMRI study of mirror reading. *Cereb. Cortex* 8 1–10. 10.1093/cercor/8.1.1 9510380

[B52] Poldrack R. A. Sabb F. W. Foerde K. Tom S. M. Asarnow R. F. Bookheimer S. Y. (2005). The neural correlates of motor skill automaticity. *J. Neurosci.* 25 5356–5364. 15930384 10.1523/JNEUROSCI.3880-04.2005 PMC6725010

[B53] Ranganathan R. Tomlinson A. D. Lokesh R. Lin T. H. Patel P. (2021). A tale of too many tasks: Task fragmentation in motor learning and a call for model task paradigms. *Exp. Brain Res.* 239 1–19. 10.1007/s00221-020-05908-6 33170341

[B54] Reis J. Schambra H. M. Cohen L. G. Buch E. R. Fritsch B. Zarahn E. (2009). Noninvasive cortical stimulation enhances motor skill acquisition over multiple days through an effect on consolidation. *Proc. Natl. Acad. Sci. U. S. A.* 106 1590–1595. 10.1073/pnas.0805413106 19164589 PMC2635787

[B55] Salmoni A. W. Schmidt R. A. Walter C. B. (1984). Knowledge of results and motor learning: A review and critical reappraisal. *Psychol. Bull.* 95:355. 6399752

[B56] Schmidt R. A. (1975). A schema theory of discrete motor skill learning. *Psychol. Rev.* 82 225–260.

[B57] Schmidt R. A. Lee T. D. (2005). *Motor learning and control: A behavioral emphasis.* Champaign, IL: Human Kinetics.

[B58] Shea C. H. Wulf G. (2005). Schema theory: A critical appraisal and reevaluation. *J. Motor Behav.* 37 85–102. 10.3200/JMBR.37.2.85-102 15730943

[B59] Shea C. H. Wright D. L. Wulf G. Whitacre C. (2000). Physical and observational practice afford unique learning opportunities. *J. Mot. Behav.* 32, 27–36. 11008269 10.1080/00222890009601357

[B60] Sherwood D. E. Lee T. D. (2003). Schema theory: Critical review and implications for the role of cognition in a new theory of motor learning. *Res. Q. Exerc. Sport* 74 376–382. 10.1080/02701367.2003.10609107 14768838

[B61] Shmuelof L. Krakauer J. W. Mazzoni P. (2012). How is a motor skill learned? Change and invariance at the levels of task success and trajectory control. *J. Neurophysiol.* 108 578–594. 10.1152/jn.00856.2011 22514286 PMC3404800

[B62] Shmuelof L. Yang J. Caffo B. Mazzoni P. Krakauer J. W. (2014). The neural correlates of learned motor acuity. *J. Neurophysiol.* 112 971–980. 24848466 10.1152/jn.00897.2013 PMC4122746

[B63] Shumway-Cook A. Woollacott M. H. (2007). *Motor control: Translating research into clinical practice.* Philadelphia: Lippincott Williams & Wilkins.

[B64] Singh H. Wulf G. (2022). Mind over body: Creating an external focus for sport skills. *Eur. J. Sport Sci.* 22 610–616. 10.1080/17461391.2021.1887367 33546575

[B65] Sorgente V. Cohen E. J. Bravi R. Minciacchi D. (2022). The best of two different visual instructions in improving precision ball-throwing and standing long jump performances in primary school children. *J. Funct. Morphol. Kinesiol.* 7:8. 10.3390/jfmk7010008 35076546 PMC8788458

[B66] Spampinato D. Celnik P. (2018). Deconstructing skill learning and its physiological mechanisms. *Cortex* 104 90–102. 10.1016/j.cortex.2018.03.017 29775838

[B67] Stefan K. Cohen L. G. Duque J. Mazzocchio R. Celnik P. Sawaki L. (2005). Formation of a motor memory by action observation. *J. Neurosci.* 25 9339–9346. 16221842 10.1523/JNEUROSCI.2282-05.2005 PMC6725701

[B68] Sternad D. (2018). It’s not (only) the mean that matters: Variability, noise and exploration in skill learning. *Curr. Opin. Behav. Sci.* 20 183–195. 30035207 10.1016/j.cobeha.2018.01.004 PMC6051545

[B69] Sternad D. Huber M. E. Kuznetsov N. (2014). “Acquisition of novel and complex motor skills: Stable solutions where intrinsic noise matters less,” in *Progress in Motor Control*, ed. Sternad D. (New York, NY: Springer), 101–124. 10.1007/978-1-4939-1338-1_8 25330888

[B70] Telgen S. Parvin D. Diedrichsen J. (2014). Mirror reversal and visual rotation are learned and consolidated via separate mechanisms: Recalibrating or learning de novo? *J. Neurosci.* 34 13768–13779. 25297103 10.1523/JNEUROSCI.5306-13.2014 PMC6608381

[B71] Thorndike E. L. Columbia University, Institute of Educational Research, Division of Psychology (1932). *The fundamentals of learning*. New York, NY: Teachers College Bureau of Publications. 10.1037/10976-000

[B72] Todorov E. Jordan M. I. (2002). Optimal feedback control as a theory of motor coordination. *Nat. Neurosci.* 5 1226–1235. 12404008 10.1038/nn963

[B73] Tseng Y. Diedrichsen J. Krakauer J. W. Shadmehr R. Bastian A. J. (2007). Sensory prediction errors drive cerebellum-dependent adaptation of reaching. *J. Neurophysiol.* 98 54–62. 10.1152/jn.00266.2007 17507504

[B74] van der Steen M. C. M. Molendijk E. B. D. Altenmüller E. Furuya S. (2014). Expert pianists do not listen: The expertise-dependent influence of temporal perturbation on the production of sequential movements. *Neuroscience* 269 290–298. 24709043 10.1016/j.neuroscience.2014.03.058

[B75] Van Horn J. D. Gold J. M. Esposito G. Ostrem J. L. Mattay V. Weinberger D. R. (1998). Changing patterns of brain activation during maze learning. *Brain Res.* 793 29–38. 9630492 10.1016/s0006-8993(98)00051-1

[B76] van Vugt F. T. Furuya S. Vauth H. Jabusch H. C. Altenmüller E. (2014). Playing beautifully when you have to be fast: Spatial and temporal symmetries of movement patterns in skilled piano performance at different tempi. *Exp. Brain Res.* 232 3555–3567. 10.1007/s00221-014-4036-4 25059908

[B77] Vassiliadis P. Derosiere G. Dubuc C. Lete A. Crevecoeur F. Hummel F. C. (2021). Reward boosts reinforcement-based motor learning. *Iscience* 24:102821. 10.1016/j.isci.2021.102821 34345810 PMC8319366

[B78] Vassiliadis P. Lete A. Duque J. Derosiere G. (2022). Reward timing matters in motor learning. *Iscience* 25:104290. 10.1016/j.isci.2022.104290 35573187 PMC9095742

[B79] Walker M. P. Brakefield T. Morgan A. Hobson J. A. Stickgold R. (2002). Practice with sleep makes perfect: Sleep-dependent motor skill learning. *Neuron* 35 205–211. 10.1016/s0896-6273(02)00746-8 12123620

[B80] Willey C. R. Liu Z. (2018). Long-term motor learning: Effects of varied and specific practice. *Vis. Res.* 152 10–16. 28688905 10.1016/j.visres.2017.03.012

[B81] Willingham D. B. (1998). A neuropsychological theory of motor skill learning. *Psychol. Rev.* 105 558–584. 9697430 10.1037/0033-295x.105.3.558

[B82] Winstein C. J. (1991). Knowledge of results and motor learning—implications for physical therapy. *Phys. Ther.* 71 140–149. 10.1093/ptj/71.2.140 1989009

[B83] Wulf G. Su J. (2007). An external focus of attention enhances golf shot accuracy in beginners and experts. *Res. Q. Exerc. Sport* 78 384–389. 10.1080/02701367.2007.10599436 17941543

[B84] Wulf G. McConnel N. Gärtner M. Schwarz A. (2002). Enhancing the learning of sport skills through external-focus feedback. *J. Motor Behav.* 34 171–182. 10.1080/00222890209601939 12057890

[B85] Wulf G. McNevin N. Shea C. H. (2001). The automaticity of complex motor skill learning as a function of attentional focus. *Q. J. Exp. Psychol. Sec. A* 54 1143–1154. 10.1080/713756012 11765737

[B86] Wulf G. Raupach M. Pfeiffer F. (2005). Self-controlled observational practice enhances learning. *Res. Q. Exerc. Sport* 76 107–111. 15810775 10.1080/02701367.2005.10599266

[B87] Wulf G. Shea C. Lewthwaite R. (2010). Motor skill learning and performance: A review of influential factors. *Med. Educ.* 44 75–84. 20078758 10.1111/j.1365-2923.2009.03421.x

[B88] Yadav G. Mutha P. K. (2016). Deep breathing practice facilitates retention of newly learned motor skills. *Sci. Rep.* 6:37069. 10.1038/srep37069 27841345 PMC5107920

[B89] Yadav G. Mutha P. K. (2020). Symmetric interlimb transfer of newly acquired skilled movements. *J. Neurophysiol.* 124 1364–1376. 10.1152/jn.00777.2019 32902352

